# Is interleukin-8 a true predictor of pediatric acute respiratory distress syndrome outcomes? Beware of potential confounders

**DOI:** 10.1186/s13054-019-2507-5

**Published:** 2019-06-27

**Authors:** Sébastien Redant, François Angoulvant, Oceane Barbance, David De Bels, Andrea Gallerani, Rachid Attou, Kianoush Kashani, Patrick M. Honore

**Affiliations:** 1 ICU Department, Centre Hospitalier Universitaire Brugmann-Brugmann University Hospital, Place Van Gehuchtenplein, 4, 1020 Brussels, Belgium; 2 Service d’accueil des urgences pédiatriques, Necker- Enfants Malades University Hospital, Assistance Publique–Hôpitaux de Paris, Paris, France; 3 Division of Nephrology and Hypertension, Division of Pulmonary and Critical Care Medicine, Mayo Clinic, Rochester, USA

We enthusiastically read the study by Flori et al. related to the potential association of interleukin-8 (IL8) with the outcomes of pediatric acute respiratory distress syndrome (PARDS) following respiratory tract infection. In this study, IL-8 found to be associated with mortality, duration of ventilation, pediatric intensive care unit (PICU) length of stay, and the degree of pulmonary morbidity, measured by oxygenation defect. Meanwhile, IL8 was not found to be associated with the occurrence of PARDS, per se [1]. Samransamruajkit et al. previously in a single-center study reported an association between mortality in PARDS and IL-8 levels (1288.7 ± 638 among non-survivors vs. 257 ± 366 pg/ml in survivors, *P* = .001) [2]. IL-8 is secreted by the lung endothelial cells in response to injury induced by infections or toxins. IL-8 concentration in bronchoalveolar lavage was found to be 5 to 10 times higher in the day of intubation when compared with control subjects. This indicates a substantial inflammation which leads to an increase of intra-alveolar neutrophils [3]. Hall et al. showed a significant association between IL-8 levels and respiratory infection with the H1N1 virus. IL8 levels in these patients were significantly higher when compared with other influenza subtypes (median of 55(13–201) vs. 0(0–13) pg/mL; *P* < .0001) [4]. A similar observation was made for *Staphylococcus aureus* in comparison with other microorganisms (164(52–4424) vs. 6.1(0–22) pg/ml; *P* = .0002) [4]. On the other hand, the PARDS-associated mortality rate is different based on the type of infection [5]. Hence, it would be reasonable to be cautious in the extrapolation of the knowledge related to PARDS-associated mortality when it is due to RSV versus PARDS-associated death in other infections [5]. Since the type of microorganism or virus influences the IL-8 level and PARDS outcome, we suggest the relationship of the IL-8 concentration and PARDS outcomes be stratified based on the type of causing microorganism or virus.

## Data Availability

Not applicable.

## Abbreviations

IL8: Interleukin-8
PARDS: Pediatric acute respiratory distress syndrome
PICU: Pediatric intensive care unit
RSV: Respiratory syncytial virus
