# Supplementary material for: Is interleukin-8 a true predictor of pediatric acute respiratory distress syndrome outcomes? Beware of potential confounders
Source: Crit Care. 2019 Jun 27;23:233. doi: 10.1186/s13054-019-2507-5 (PMC6595614; doi:10.1186/s13054-019-2507-5)
Supplement: Related texts

# Reply: Authors’ response

**Authors:** Heidi Flori, Mary K. Dahmer

We appreciate the insightful commentary by Dr. Redant and colleagues on our publication demonstrating that IL-8 is associated with poor outcome in children with severe acute respiratory failure, many of which had pediatric acute respiratory distress syndrome (PARDS) [1]. Indeed, it is important for research to generate the “next questions” to investigate. This is particularly true for understanding which biological markers may be impacted by individual disease processes, types of infection, host response characteristics, etc. Such understanding is the impetus behind the “precision medicine” movement.

Dr. Redant et al. suggest that analysis of the relationship between IL-8 and outcomes be stratified by type of microorganism causing infection. However, as an ancillary study to a sedation management trial, we do not have detailed infectious agent data. As shown in the supplement, median IL-8 levels varied by primary diagnosis; sepsis patients had a higher median IL-8 than those with pneumonia or bronchiolitis. Although not reported in the paper, IL-8 is independently associated with the duration of mechanical ventilation (*P* < 0.0005, all days) after adjusting for age, PRISM-III, and primary diagnoses (pneumonia [37%], bronchiolitis [20%], sepsis [19%], asthma [10%], aspiration pneumonia [6%], and other [8%]). Since the group with sepsis likely has a higher proportion of bacterial infections, and those with bronchiolitis likely have a high proportion with viral infections, one might speculate that IL-8 is associated with poor outcome independent of a bacterial or viral etiology. However, analysis of microorganism-based subgroups would be a much “cleaner” way to examine that question, yet such a strategy is hampered by the size of pediatric cohorts. Although the BALI study is one of the largest multi-center cohorts of pediatric acute respiratory failure and PARDS patients, our subgroup analyses still suffered from low numbers, especially when adjusting for important covariates. We anticipate this is also true for most other PARDS cohorts.

In summary, we agree there is value in stratifying analyses of IL-8 and outcomes by an infectious agent, which would demonstrate whether IL-8 is associated with outcome irrespective of underlying etiology, or, alternatively, varies by infectious agent. The use of a common set of data elements in PARDS-related studies may allow this question to be addressed in the future by combining datasets from multiple studies. Nonetheless, IL-8 remains an intriguing biomarker of inflammation in critical illness where it may be useful for stratification into endotypes [6] allowing predictive and prognostic enrichment for trials, and/or as a marker of disease trajectory with regard to response to therapy [7].
